# Correlation between Trabecular Bone Score and Homocysteine Level in Rheumatoid Arthritis Patients on Anti-TNF Inhibitors

**DOI:** 10.3390/life14040463

**Published:** 2024-04-01

**Authors:** Florentina Ioniță-Radu, Iulia-Nadine Nicolau, Oana-Georgiana Petrache, Maria-Laura Groșeanu, Violeta-Claudia Bojincă, Maria-Magdalena Negru, Sandica Bucurică, Daniela Anghel

**Affiliations:** 1 Department of Gastroenterology, Dr. Carol Davila Central Military Emergency University Hospital, 010825 Bucharest, Romania; 2 Department of Gastroenterology, Carol Davila University of Medicine and Pharmacy, 020021 Bucharest, Romania; 3 Department of Internal Medicine 2, Dr. Carol Davila University Central Military Emergency Hospital, 010825 Bucharest, Romania; 4 Department of Rheumatology, Carol Davila University of Medicine and Pharmacy, 020021 Bucharest, Romania; 5 Department of Rheumatology, ‘Sf. Maria’ Clinical Hospital, 010024 Bucharest, Romania; 6 Department of Internal Medicine, ‘Sf. Maria’ Clinical Hospital, 010024 Bucharest, Romania; 7 Department of Medico-Surgical and Prophylactic Disciplines, Titu Maiorescu University, 031593 Bucharest, Romania

**Keywords:** rheumatoid arthritis, trabecular bone score, bone mineral density, homocysteine, 25-OH vitamin D, anti-TNF α agents

## Abstract

Rheumatoid arthritis (RA) is an independent osteoporosis risk factor. Biologic and immunosuppressive treatment, and levels of homocysteine and 25-OH vitamin D may influence the trabecular bone score (TBS) in RA patients. We aimed to compare the effects of biological (b) and conventional synthetic (cs) disease-modifying anti-rheumatic drugs (DMARDs) on TBS in patients with RA and hyperhomocysteinemia (HHcy) or 25-OH vitamin D deficiency. Patients who had tests conducted for trabecular bone score, bone mineral density (BMD), homocysteine (Hcy) and 25-OH vitamin D at an interval of one year and met the inclusion criteria were enrolled in this retrospective study. Sixty-four patients with RA were enrolled and were divided into the following two groups: the first group (34 patients) had received treatment with bDMARDs and the second group (30 patients) had received csDMARDs. BDMARDs and csDMARDs had a positive influence on TBS and BMD. The best results were observed in the Adalimumab group (*p* = 0.033). Hyperhomocysteinemia and 25-OH vitamin D deficiency led to lower TBS values. Both bDMARDs and csDMARDs positively affected TBS and BMD in RA patients. High homocysteine serum levels or 25-OH vitamin D deficiency had a negative impact on TBS and BMD after 12 months. Our study aims to show the potential benefits of anti-TNF α drugs on TBS. This impact appears to be strongly associated with serum 25-OH vitamin D and homocysteine levels. Anti-TNF drugs may increase bone mineral density and microstructure. As a result, they may minimize the incidence of fractures in RA patients.

## 1. Introduction

Rheumatoid arthritis is a chronic inflammatory disease that most commonly affects the small joints of the hands and feet, leading to progressive destruction of joints and extra-articular complications. RA is one of the typical independent risk factors for secondary osteoporosis (OP). Decreased bone mass and bone density are caused by several factors involved in the pathogenesis of RA, such as the presence of pro-inflammatory cytokines (tumor necrosis factor-TNFα, Interleukin-1-IL-1, Interleukin-6-IL-6) and chronic systemic inflammation, which impacts bone metabolism [1]. Moreover, a decrease in bone mass is seen in RA patients because of other factors such as the predisposition for the female gender, older age, disability or immobility secondary to the disease activity, menopausal state, and use of glucocorticoids (GC). RA patients have a 33% increased risk of fractures compared to healthy subjects [2]. Pro-inflammatory cytokines stimulate osteoclast activity via the receptor activator of nuclear factor kappa-Β ligand (RANK-L). Thus, the degree of disease activity is directly associated with the development of OP. On the other side, GC is often used in the treatment of RA, which can determine bone loss by the following two mechanisms: first, by the inhibitory effect on bone formation, and second, by increasing bone resorption due to an improper function of the RANK-L/osteoprotegerin (OPG) system [3].

Osteoporosis is a frequent complication of RA, affecting twice as many people as the general population. OP is a common systemic disease characterized by bone fragility and fractures due to decreased bone mineral density (BMD). BMD can be measured by using a technique called dual-energy X-ray absorptiometry (DXA), which indicates the risk of fracture according to T score (T < −2.5 indicates OP). Although BMD measurement is the standard investigation for diagnosing OP, this tool does not evaluate the bone quality represented by bone microarchitecture [1,4]. Recently, a new tool has been studied for evaluating the trabecular bone in addition to BMD or as an independent method: the trabecular bone score (TBS). TBS is a new measurement represented by a texture index from lumbar spine DXA images, which indirectly evaluates the bone microarchitecture. If TBS has a low value, this indicates a weaker bone and a higher fracture risk [5]. Besides being non-invasive, TBS is an efficient method for predicting fracture risk. It has been studied particularly in secondary causes of OP such as rheumatoid arthritis, hyperparathyroidism, and long-term glucocorticoid (GC) exposure [4,5]. Another difference between BMD and TBS is that the first tool has limitations in predicting the fracture risk in rheumatoid arthritis, which has been demonstrated by normal T scores in patients with fractures [5]. Therefore, TBS is superior to BMD for assessing the risk of vertebral fractures in RA patients. 

Other risk factors are associated with an increased risk of OP and consecutive fractures in RA patients, such as low 25-OH vitamin D levels, sedentarism, and long-term use of GC. Casabella et al. showed that patients with RA have lower 25-OH vitamin D levels and lower TBS scores in the lumbar spine compared with healthy subjects. Therefore, RA patients have a higher risk for OP and fragility fractures [6,7]. Vitamin D is essential in calcium metabolism and bone mineralization and has immunomodulatory effects. Studies have demonstrated that a low 25-OH vitamin D level may be a risk factor for autoimmune diseases [2]. Low vitamin D concentrations influence bone turnover and have a negative impact on bone architecture, thereby impacting TBS. However, it is known that vitamin D has a predominant effect on cortical bone rather than the trabecular one [8].

Studies have demonstrated that anti-TNF agents may play a role in TBS changes, particularly in the presence of low levels of 25-OH vitamin D. TNF inhibitors may prevent bone loss and a correlation between the degree of inflammation and BMD values was established. High C reactive protein levels (CRP) were associated with low BMD values. Therefore, biological treatment was associated with decreased bone loss [9]. On the contrary, Toussirot et al. showed that TBS decreased in RA patients while they were on anti-TNF therapy [7].

In addition to vitamin D and calcium, other nutrients, such as homocysteine (Hcy) and B group vitamins, are also involved in bone metabolism. Homocysteine is a non-essential amino acid whose values can be increased in RA, particularly secondary to Methotrexate therapy. Methotrexate inhibits folic acid; therefore, a low folic acid level leads to hyperhomocysteinemia due to decreased Hcy catabolism. Other causes for HHcy are excessive methionine intake or genetics [10].

**Figure 1:**
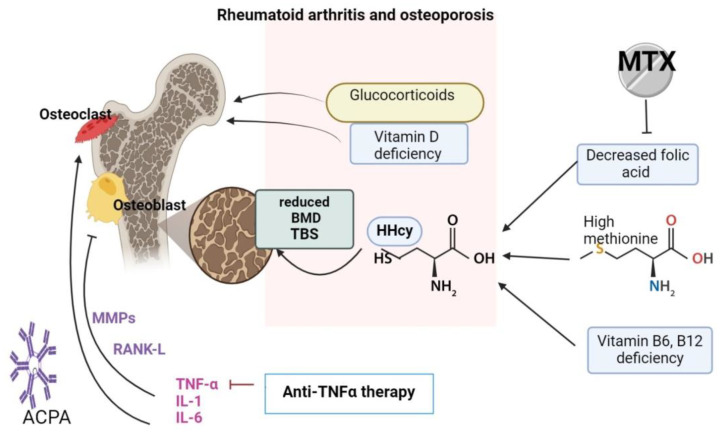
Hyperhomocysteinemia-induced bone damage mechanism (created with BioRender).

Hcy has been shown to play an important role in RA’s complications, is a well-known risk factor for cardiovascular diseases and has also shown some implications in bone metabolism [10]. Hyperhomocysteinemia can lead to reduced bone mineral density (BMD) and changes in microarchitecture (Figure 1), influencing TBS and increasing bone fragility, resulting in a higher risk of fractures. These alterations appear as a result of increased osteoclast activity and bone resorption, to the detriment of bone formation [11].

Vitamin B6, B12 and B9 (folic acid) deficiency or dietary methionine excess can cause hyperhomocysteinemia (HHcy). Folic acid can be particularly decreased in rheumatoid arthritis patients because of Methotrexate therapy. Methotrexate (MTX) therapy (a folic acid antagonist) can cause folic acid deficiency.

Glucocorticoid therapy and vitamin D deficiency favor the occurrence of osteoporosis.

Proinflammatory cytokines are increased in RA and can cause bone damage through matrix metalloproteinases (MMPs) activation and RANKL expression, which promote osteoclastogenesis and bone resorption (resulting in reduced bone mineral density (BMD) and a reduced trabecular score (TBS).

Anti-citrullinated protein antibodies (ACPA) can also stimulate osteoclast formation and promote bone damage.

In RA patients, osteopenia appears as both periarticular and systemic, and the mechanisms are quite similar: the pro-inflammatory cytokines (TNF-α, IL-6, IL-1) and systemic inflammation, as previously mentioned, together with the RANK-L, have an inhibitory effect on osteoblastogenesis and a stimulatory effect on osteoclastogenesis, which results in lower BMD and TBS. Periarticular bone loss in RA results from cortical bone thinning at the insertion site of the inflamed synovial membrane, bone trabeculae reduction, and increased cortical porosity. Both systemic and local bone loss appear in the pre-clinical stage of RA. In addition, the presence of ACPA (anti-citrullinated peptides antibodies) is associated with an increased risk for osteopenia, particularly in the metacarpal area, as a consequence of the direct action of ACPA on osteoclasts, via IL-8 [12]. Moreover, high serum levels of Dickkopf-related protein 1 (Dkk-1), a Wnt (wingless-related integration site)-signaling inhibitor with essential effects on the bone system, are found in RA patients [13].

To sum up, this study aimed to assess the trabecular bone score changes in rheumatoid arthritis patients with high homocysteine serum levels and hypovitaminosis D and to establish the potential effect of anti-TNF agents-bDMARDs and csDMARDs on TBS.

To our knowledge, there are very few data in the literature evaluating the beneficial role of this therapy on trabecular bone score in rheumatoid arthritis patients with hyperhomocysteinemia or vitamin D deficiency. Therefore, more studies are needed in order to clarify whether biologics have a positive and direct impact on bone cells or an indirect effect by lowering the disease activity and consecutively improving mobility. Moreover, future research is needed to establish the correlation between anti-TNF therapy, Hcy levels, vitamin D deficiency, and bone metabolism in patients with rheumatoid arthritis.

## 2. Materials and Methods

### 2.1. Study Population

**Figure 2:**
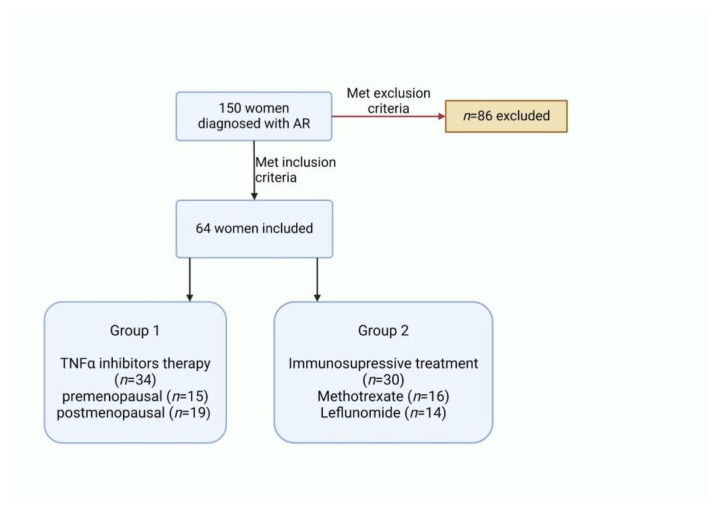
The study population divided into groups.

We performed a retrospective observational study that included 64 females (150 were considered, but 86 were excluded) diagnosed with rheumatoid arthritis (Figure 2) according to the 2010 American College of Rheumatology/European League Against Rheumatism classification criteria for rheumatoid arthritis [14]. The patients were enrolled in this study after obtaining written informed consent for data collection during routine clinical assessment in our department (“Carol Davila” Central Emergency Military University Hospital, Bucharest, Department of Internal Medicine 2). The data were collected in chronological order from patients who were hospitalized from June 2022 to June 2023.

The recruited patients were treated according to the EULAR recommendations for the management of RA [15]. They were divided into two groups: the first group of 34 patients was treated with anti-TNF alpha drugs (Adalimumab, Etanercept, Infliximab) and the second group of 30 patients was treated with immunosuppressant agents (Leflunomide and Methotrexate).

The inclusion criteria were as follows: patients over 18 years old, female gender, diagnosed with RA (according to ACR/EULAR 2010 criteria), who had DXA assessment at an interval of 12 months before and after csDMARDs/bDMARDs initiation as well as serum level testing for Hcy, 25-OH Vitamin D, and inflammatory markers. 

The exclusion criteria were as follows: anti-osteoporotic treatment (vitamin D supplementation, bisphosphonates, selective modulators of estrogen receptors, biologic therapy), secondary causes of osteoporosis (thyroid or parathyroid disorders, diabetes mellitus), rheumatic inflammatory diseases other than RA or disorders known to interfere with bone turnover, long-term or high-dose glucocorticoid therapy, and history of biologic treatment.

Demographic data and a complete medical history, including age, female gender, medication history, comorbidities, osteoporosis risk factors, and history of vertebral fractures, were collected (Table 1). All patients had laboratory analyses and osteodensitometry performed at an interval of 12 months. To minimize biases, patients were selected in a consecutive manner, and we used clear criteria in selecting patients.

The study protocol was approved by the local ethics committee (No 602/28 June 2023).

### 2.2. Group Analysis

A number of 64 females (34 in the first group on anti-TNF alpha drugs and 30 on immunosuppressant agents in the second one) with rheumatoid arthritis were included in this study.

We compared the data for each group using the Student’s *t*-test method at baseline and after 12 months of treatment. The T score and TBS were compared between the two groups, between the baseline values and after 12 months of treatment. Correlation analyses were performed between TBS, T score, serum 25-OH vitamin D and homocysteine levels, and DAS-28 (disease activity score) related to other parameters: the presence of RF (rheumatoid factor), ACPA, and age.

The first group included 34 females (15 premenopausal and 19 postmenopausal females) treated with TNFα inhibitors (Adalimumab, Etanercept, and Infliximab) and were classified according to age and values of the following parameters: RF, ACPA, TBS, T score, 25-OH vitamin D, homocysteine, and DAS-28.

The second group included 30 patients treated with immunosuppressive agents (16 treated with Methotrexate and 14 with Leflunomide) who were also classified according to age and values of the same parameters: RF, ACPA, TBS, T score, 25-OH vitamin D, homocysteine and the DAS-28 score.

### 2.3. Clinical and Laboratory Evaluation

Laboratory assessments included a complete blood count, serum C-reactive protein (CRP) using a Beckman Coulter AU5822/U.S analyzer (Beckman Coulter, Chaska, MN, USA)), erythrocyte sedimentation rate (ESR), liver and renal function tests, serum albumin, thyroid-stimulating hormone and free T4 levels, serum rheumatoid factor (using a Beckman Coulter/U.S analyzer), anti-cyclic-citrullinated peptide antibodies (ACPA-using ORGENTEC Alegria (Mainz, Germany)), homocysteine levels-chemiluminescent microparticle immunoassay (Architect Homocysteine assay, Abbott analyzer (Abbott Park, IL, USA) and urinary analysis. 

Bone parameters, including serum calcium, phosphate, alkaline phosphatase, parathormone, and 25-OH vitamin D levels, were also evaluated. Serum homocysteine levels were also classified according to the reference range as follows: normal values between 5.46–16.20 μmol/L, hyperhomocysteinemia >16.20 μmol/L.

25-OH vitamin D serum levels were determined using an Abbott Alinity/U.S. analyzer. 25-OH vitamin D serum levels were classified as deficiency (<20 ng/mL), insufficiency (20 to 30 ng/mL), and optimal values ≥ 30 ng/mL [16,17,18]. To ensure the accuracy and reliability of these measurements, we ensured that the samples were processed by trained and certified laboratory personnel who follow standardized procedures and protocols and adhere to regulator guidelines and standards. 

The disease activity score 28 (DAS-28) was also evaluated. This score is used for assessing the disease activity in rheumatoid arthritis patients and is categorized as follows: high disease activity as DAS-28 >5.1, moderate disease activity as >3.2–5.1, low disease activity as 3.2–2.6, and remission as DAS-28 ≤2.6 [19]. 

### 2.4. Bone Mineral Density (BMD)

Dual X-ray absorptiometry (Lunar IDXA, GE Medical Systems, Madison, WI, USA) was used to evaluate the BMD (g/cm^2^) for all patients included in our study. The lumbar spine (L1–L4) and left and right femur were assessed to obtain DXA scans following the manufacturer’s instructions. The database used as a reference for the spine and left femur matched the age, sex, weight, and ethnicity.

To determine BMD values represented by a T score, several variables, such as weight, height, age, ethnicity, and gender, were used. The T score reflects the degree of bone mineralization. According to the World Health Organization (WHO), a T score at/above −1.0 SD (standard deviation) is considered normal, a T score between −1.0 and −2.5 SD is defined as osteopenia and a value at/below −2.5 SD is classified as osteoporosis. It is essential to mention that the same examiner acquired all the DXA images before and after 12 months.

### 2.5. Trabecular Bone Score (TBS)

TBS, another critical parameter represented by a texture index from a DXA image, was also used to assess the bone microarchitecture. 

TBS (version: 3.0.0.15) was extracted from each lumbar spine DXA examination using the TBS index (iNsight software V3.0).

TBS reflects Parfitt’s microarchitecture parameters as follows: number of trabeculae, trabecular separation, trabecular bone volume, and connectivity density [20]. 

Trabecular bone is the main type of bone tissue in vertebrae; therefore, TBS is derived from an L1-L4 lumbar spine DXA image. Moreover, the region analyzed for TBS is the same region used for BMD. Thus, the vertebrae excluded from BMD (the fractured ones) are also excluded from TBS [21].

Low values of TBS are associated with a weaker bone and a higher risk of fractures; therefore, TBS ≥1.350 is considered a standard value, a TBS 1.200–1.350 is defined as partially degraded microarchitecture, and TBS ≤1.200 is classified as degraded microarchitecture [22].

### 2.6. Statistical Analysis

Statistical analysis was performed using Statistical Package for Social Sciences (SPSS) software, version 20 (IBM Corp., New York, NY, USA), and the following statistical tools: chi-square test, Student’s t-test and ANOVA repeated measures. The chi-square test (χ^2^ test) was used to analyze categorical data in order to evaluate the association between parameters using data from both groups (64 patients—34 in the first group and 30 in the second group). A verification test of the initial values and after 12 months was generated using Bonferroni correction.

To compare the two groups, we used the Student’s *t*-test method. The following *p* values were accepted: *p* < 0.05 significant in a confidence interval (CI) of 95%, *p* < 0.01 significant (CI of 99%), *p* < 0.001 highly significant (CI of 99.9%).

The results were presented as mean, standard deviation, median, and range.

## 3. Results

### 3.1. Assessment of BMD, TBS, 25-OH Vitamin D, and Homocysteine Serum Levels in the First Group

The first group included 34 females (15 premenopausal and 19 postmenopausal) treated with TNFα inhibitors (Adalimumab, Etanercept, and Infliximab) and were classified according to age and the values of the following parameters: RF, ACPA, TBS, T score, 25-OH vitamin D, homocysteine and DAS-28. 

The serum level of homocysteine was normal for 16 patients (47.1%) and increased in 18 patients (52.9%). A high level was found in 11 postmenopausal women (73.3%) and in 7 premenopausal women (57.8%). Using the T score values at baseline (T1 score), we classified the patients as follows: 6 patients (17.6%) had a normal T1 score, and 28 patients presented with abnormal values (14 women had osteopenia-41.2% and the other 14 women had osteoporosis-41.2%. 

Among premenopausal women, six had a normal T1 score-40%, one patient had osteoporosis-6.66%, and eight had osteopenia-53%. Among postmenopausal women, osteoporosis was found in 13 patients-68.4% and osteopenia in 6 patients-31.6%. Lumbar spine TBS at baseline (TBS 1) was normal in 2 patients (5.9%), and for the remaining 32 patients (94.1%), TBS1 had a low value. Nineteen patients (55.8%) had a slight bone architectural change, and 13 patients (38.2%) had a significantly low TBS1 value. 

Among postmenopausal women, we found a significantly modified TBS1 value in 11 patients (55.9%) and a slightly modified TBS1 in 8 women (42.1%). 

Among premenopausal women, only 2 (13.3%) had a significantly reduced TBS1 value, 11 women (73.3%) had a slightly modified TBS1, and normal values were found in 2 patients (13.3%). The serum 25-OH vitamin D level was normal in 14 patients (41.2%), and 20 patients (58.8%) had 25-OH vitamin D insufficiency (<20 ng/mL). Among premenopausal women, a low 25-OH vitamin D level was found in 8 cases (53.3%); among postmenopausal women, 25-OH vitamin D insufficiency was found in 12 cases (63.1%). In this group, 12 patients were treated with Etanercept (35.3%), 9 were treated with Infliximab (26.5%), and 13 patients were treated with Adalimumab (38.2%). (Table 2 and Table 3).

In the first group (biological disease-modifying anti-rheumatic drugs-bDMARDs), the T score improved in patients with osteopenia and osteoporosis after 12 months. Among these patients, only 17.64% had no change in T score. Most patients had an improved BMD: 58.82% presented a favorable evolution with 1 unit, respectively, and 23.52% showed a positive evolution, with 2 units. In patients with normal BMD at baseline, we did not report any modification after 12 months (Table 2).

In patients with low serum 25-OH vitamin D levels, we observed that 85% had a normal T2 score and 15% had osteopenia. 

Among the bDMARDs group, 11 patients (32.3%) showed an improved TBS value after 12 months of treatment, and 21 patients (61.7%) presented no change in TBS2 after 12 months (Table 4).

We observed a highly significant correlation (*p* < 0.0001) between low T scores and low 25-OH vitamin D levels at baseline, and also a highly significant correlation (*p* = 0.0001) between improved T2 scores and optimal 25-OH vitamin D levels after 12 months.

TBS was not correlated with 25-OH vitamin D levels at baseline (*p* = 0.1), but we found a significant correlation (*p* = 0.02) between TBS2 and 25-OH vitamin D levels after 12 months.

Modified TBS1 was correlated (*p* < 0.0002) with high homocysteine levels at baseline. After 12 months of anti-TNFα treatment, modified TBS was significantly correlated (*p* < 0.0001) with hyperhomocysteinemia.

### 3.2. Assessment of BMD, TBS, 25-OH Vitamin D, and Homocysteine Serum Levels in the Second Group

The second group included 30 patients treated with immunosuppressive agents (16 treated with Methotrexate and 14 with Leflunomide) who were also classified according to age and values of the following same parameters: RF, ACPA, TBS, T score, 25-OH vitamin D, homocysteine, and DAS-28 score. 

The second group included 12 females aged between 20–50 years—the premenopausal women subgroup (40%) and 18 females aged >50 years—the postmenopausal subgroup (60%). Six patients (20%) had a normal value of the ACPA and RF (seronegative rheumatoid arthritis), and 24 patients (80%) had seropositive rheumatoid arthritis (positive RF ± ACPA). 

The serum level of homocysteine was normal in 11 patients (36.7%) and increased in 19 patients (63.3%). The T score at baseline (T1 score) was classified as follows: a total of 7 patients (23.3%) had a normal T1 score, 11 patients (36.6%) had osteopenia, and osteoporosis was present in 12 patients (40%). 

Lumbar spine TBS at baseline (TBS 1) was normal in six patients (20%). In 24 patients, a low TBS1 value was noted, from which 14 patients (46.6%) were reported to have a slight bone architectural change, and 10 patients (43.7%) showed a significantly low TBS1 value. The serum 25-OH vitamin D level was normal in 8 patients (26.7%), and 22 patients (73.3%) had 25-OH vitamin D insufficiency (<20 ng/mL). 

In the second group, 16 patients were treated with Methotrexate (53.3%) and 14 with Leflunomide (46.7%). Regarding the T score after 12 months of treatment, a favorable evolution was observed in 5 patients (16.66%), no change was noticed in 11 patients (36.66%), and a worsening status was observed in 14 patients (46.66%). In women between 20 and 50 years old (40% of this group), the T2 score had normal values in 16.67% of patients, osteopenia in 58.33%, and osteoporosis in 25% of cases after 12 months. The following results were observed in women above 50 years old (60% of this group): T2 score was normal in 5.55% of patients, 55.55% had osteopenia, and 38.88% had osteoporosis. 

In patients with hyperhomocysteinemia, 63.15% had lower T2 score values after 12 months.

No change in TBS2 was observed in 47.36% of patients with hyperhomocysteinemia after 12 months. (Table 5, Table 6 and Table 7).

### 3.3. Comparison of the TBS, BMD, Hcy, and 25-OH Vitamin D between RA Patients from the bDMARDs Group

Correlation analyses were performed between homocysteine concentrations and TBS 2, T2 score, 25-OH vitamin D serum levels, and DAS-28 for both groups. 

In other words, we observed a series of changes in the bone metabolism of RA patients after 12 months of follow-up.

**Figure 3:**
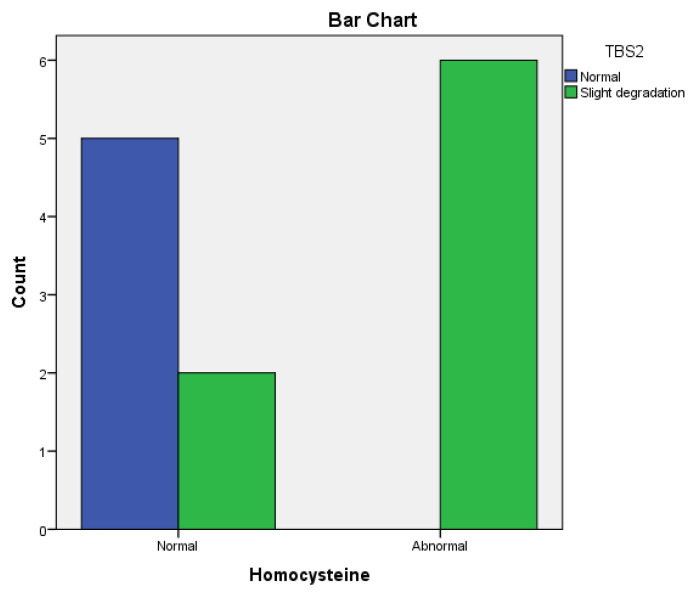
The influence of Hcy on TBS values in RA patients treated with Adalimumab.

In the bDMARDs-treated group, we observed a significant correlation between TBS at baseline and TBS after 12 months (r = 0.59, *p* = 0.033) in patients treated with Adalimumab (Figure 3). However, no significant correlation was found in patients treated with Etanercept (r = 0.54, *p* = 0.065) and Infliximab (r = 0.50, *p* = 0.170). 

**Figure 4:**
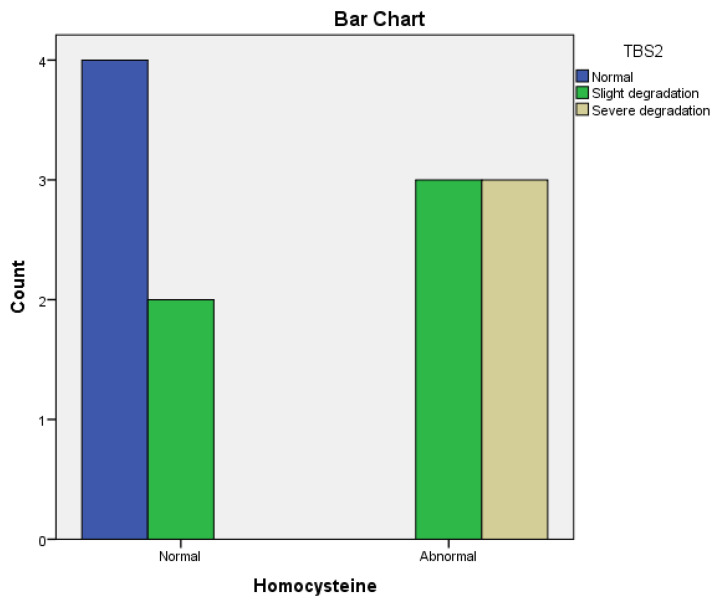
The influence of Hcy on TBS values in RA patients treated with Etanercept.

A significant correlation was also observed between TBS and hyperhomocysteinemia in both the Adalimumab- (r = 0.732, *p* = 0.004) (Figure 3) and Etanercept-treated (r = 0.76, *p* = 0.004) groups (Figure 4) after 12 months. No significant correlation was found in the Infliximab-treated group (r = 0.47, *p* = 0.197). 

**Figure 5:**
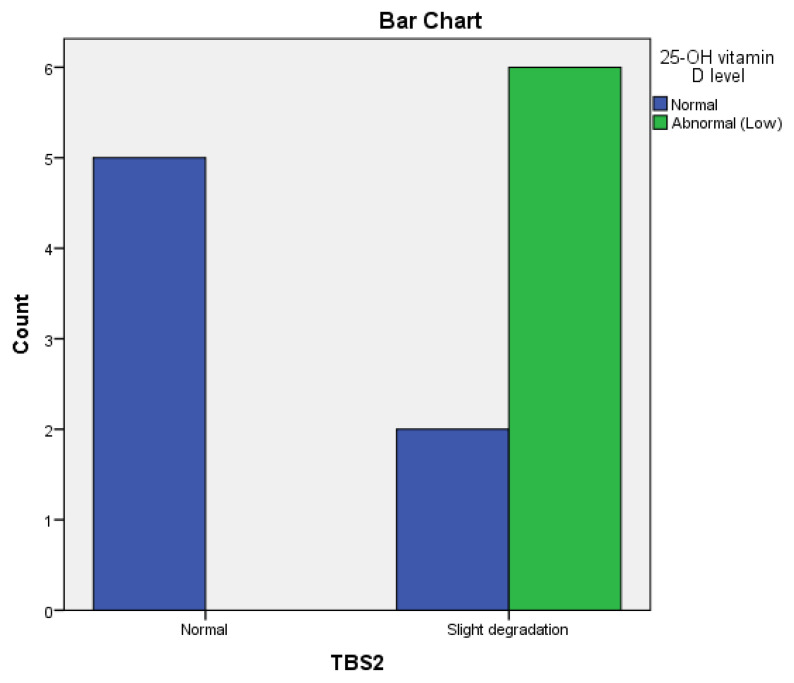
The influence of 25-OH vitamin D deficiency on TBS values in RA patients treated with Adalimumab.

In the bDMARDs group, a significant correlation (*p* = 0.0001) was found between a lower BMD and hyperhomocysteinemia at baseline and after 12 months of treatment. Regarding 25-OH vitamin D serum levels at baseline and improved TBS2, a positive correlation was found in the Adalimumab group (r = 0.73, *p* = 0.004) (Figure 5). However, no significant correlation was noticed in the Infliximab group (r = 0.50, *p* = 0.17) or in the Etanercept group (r = 0.38, *p* = 0.213) after 12 months. 

A significant correlation was also observed between improved BMD and 25-OH vitamin D levels in the Adalimumab (*p* = 0.0003) and Etanercept groups (*p* = 0.0001) but not in the Infliximab group (r = −0.50, *p* = 0.170) after 12 months, compared to baseline. 

We did not find a significant correlation between TBS2 and DAS-28 (r = 0.23, *p* = 0.443) nor between T2 scores and DAS-28 after 12 months in the Adalimumab group. We also found no significant correlation between DAS-28 and TBS2 in the Etanercept (r= −0.12, *p* = 0.71) and Infliximab groups (r = 0.47, *p* = 0.197).

### 3.4. Comparison of the TBS, BMD, Hcy, and 25-OH Vitamin D between RA Patients from the csDMARDs Group

**Figure 6:**
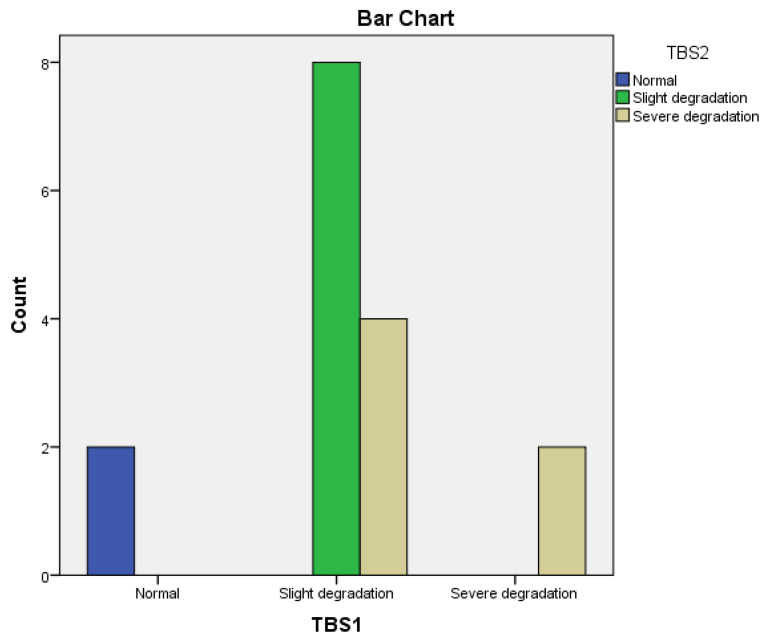
The correlation between TBS at baseline and TBS after 12 months in the Methotrexate-treated group.

A significant correlation was observed between TBS at baseline and TBS after 12 months in the Methotrexate-treated group (r = 0.75, *p* = 0.001) (Figure 6) and the Leflunomide group (r = 0.52, *p* = 0.05).

We observed a significant correlation between improved BMD (T2 score) and normal homocysteine serum levels in Leflunomide-treated patients (r = 0.71 *p* = 0.004); however, no correlation was observed between TBS2 and homocysteine (r = 0.48, *p* = 0.07) in these patients after 12 months of treatment. 

**Figure 7:**
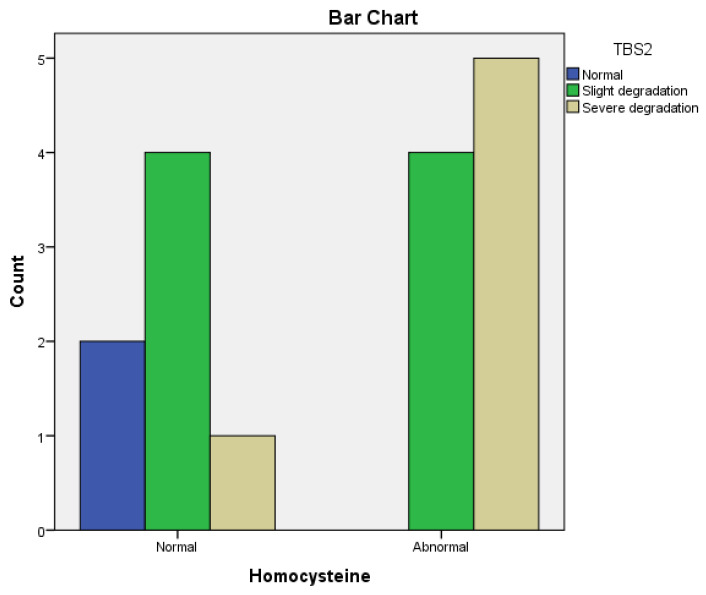
The correlation between improved TBS2 and normal homocysteine serum levels in the Methotrexate group.

In the Methotrexate group, we noticed a strong positive correlation between improved TBS2 and normal homocysteine serum levels (r = 0.52, *p* = 0.0003) (Figure 7) and a significant correlation between BMD (T2 score) and homocysteine levels (r = 0.31, *p* = 0.0002) after 12 months of treatment.

**Figure 8:**
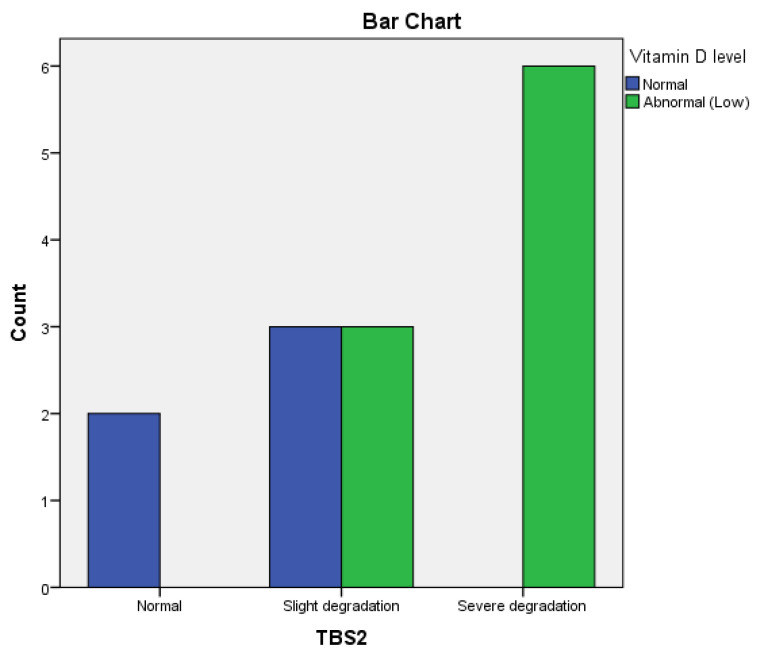
The influence of 25-OH vitamin D deficiency on TBS values in RA patients treated with Leflunomide.

A significant correlation was found between 25-OH vitamin D serum levels and TBS after 12 months of treatment in patients treated with Methotrexate (r = −0.65, *p* = 0.006). Still, no association was found between 25-OH vitamin D and BMD in the same group (r = −0.34, *p* = 0.19). In the Leflunomide-treated group, we also found a significant correlation between 25-OH vitamin D and TBS (r = 0.73, *p* = 0.003) after 12 months of treatment (Figure 8). Still, no significant correlation between BMD and 25-OH vitamin D (r = 0.51, *p* = 0.058) was found after 12 months.

Furthermore, a significant correlation was observed regarding BMD and DAS-28 (r = 0.52, *p* = 0.035) in the Methotrexate-treated group, but no significant correlation between TBS2 and DAS-28 after 12 months was observed (r = 0.15, *p* = 0.57). On the contrary, no significant correlation was observed between the DAS-28 score and BMD (r = 0.38, *p* = 0.178), nor between the TBS and DAS-28 score (r = 0.38, *p* = 0.17) in the Leflunomide-treated group after 12 months. 

## 4. Discussion

Rheumatoid arthritis is characterized by systemic inflammation (high levels of proinflammatory cytokines–TNF, IL-1, IL-6, matrix metalloproteinases-MMP, circulating autoantibodies) that disrupt bone metabolism. Therefore, RA is one of the most important causes of secondary osteoporosis (the prevalence of osteoporosis is twice that of individuals without RA in the same age group) [1].

In this retrospective study, we evaluated the influence of biological and conventional synthetic DMARDs on BMD and TBS in rheumatoid arthritis patients.

In our study, we found a positive correlation between anti-TNFα agents (Adalimumab, Etanercept, and Infliximab) and improvements in the T score and TBS. 

TBS and BMD were significantly improved after 12 months of treatment in the first group. The best results were found in patients who were treated with Etanercept and Adalimumab; the majority of patients who were treated with Etanercept showed better T score results, and half of them demonstrated a better TBS value (the other half showed a stationary status). Furthermore, patients who received Adalimumab showed the best results on TBS after 12 months. 

Other studies have demonstrated the beneficial effect of bDMARDs on TBS in rheumatoid arthritis patients, especially in premenopausal females. In a prospective study, Killinger et al. showed i that biologic therapy led to an increase in TBS values and osteocalcin after 12 months of follow-up compared to csDMARDs. In contrast to our results, Killinger showed no effect of bDMARDs on BMD [23]. 

This positive effect of anti-TNF α drugs on bone metabolism is not surprising, given that inflammatory cytokines (TNF, IL-1, IL-6) and chronic systemic inflammation are directly related to the decrease in bone mass and bone density [12,24]. Biologic DMARDs suppress the proinflammatory cytokines and the production of MMP and stop the receptor activator of nuclear factor kappa beta (RANKL) expression, thereby decreasing the deterioration of bone density [25]. Research has shown that bDMARDs have a protective role against bone loss. TNFα inhibition reduces bone loss by limiting the activation of mature cells and the proliferation of osteoclast precursors, as demonstrated by several convincing studies conducted in animal models. Moreover, TNFα suppression decreased both inflammation and bone loss in murine collagen-induced arthritis [26].

Our study found a significant correlation between different csDMARDs and bone metabolism. We observed that RA patients on Leflunomide therapy had better DMO values after one year compared to patients treated with Methotrexate.

Kwon et al. observed that, among the csDMARDs, Leflunomide can improve lumbar spine BMD in RA patients with osteoporosis [27]. Methotrexate, the gold-standard treatment in rheumatoid arthritis, can indirectly reduce bone destruction by suppressing synovitis. On the other hand, in high doses, it can change bone metabolism, decreasing osteoblast differentiation and the production of bone matrix [28]. Our study found no correlation between TBS2 and disease activity in the csDMARDs group.

Other studies have demonstrated the importance of TBS in evaluating the risk of fractures in rheumatoid arthritis patients as a complementary tool to BMD and FRAX [29]. Choi et al. showed that TBS has a discriminatory role for osteoporotic fractures in rheumatoid arthritis females who reached the stage of menopause due to the low TBS values in patients with vertebral fractures and normal BMD values [30]. 

Regarding homocysteine, we found a correlation between lower TBS and low Hcy serum levels at baseline and after 12 months in the Adalimumab- and Etanercept-treated subgroups. 

Moreover, in the premenopausal females treated with Methotrexate, a correlation was reported between TBS 2 and Hcy after one year.

Studies have shown that high Hcy levels lead to lower TBS and BMD due to an effect on osteoclast activity. RA patients have an increased level of Hcy primarily because of Methotrexate treatment, which inhibits folic acid, resulting in low Hcy catabolism. The 677C_T polymorphism of the MTHFR gene is involved in bone metabolism and hyperhomocysteinemia. Vitamin B intake (including vitamin B9-folate) may positively affect bone health in patients with this polymorphism [11]. Vitamin B intake (including vitamin B9–folate, taken by patients on Methotrexate regimen) mediates the methionine metabolism. Deficiency in these vitamins can lead to an increased Hcy serum level and poor bone quality [31].

In the bDMARDs group, we observed a significant correlation between TBS and Hcy after one year of treatment with Adalimumab and Etanercept, demonstrating this metabolite’s role in changing bone microarchitecture.

Herrmann et al. showed that Hcy interferes with bone metabolism through several pathways, as follows: stimulation of osteoclasts activity, inhibition of osteoblasts activity, decrease in blood flow to the bone system; and alteration to the bone matrix by a direct effect of Hcy [32]. High Hcy levels lead to bone matrix alteration due to increased osteoclast activity by activating matrix metalloproteinases (MMP) and increasing oxidative stress due to decreased nitric oxide levels [31,33].

In our study, we observed a significant correlation between 25-OH vitamin D serum levels and BMD and between 25-OH vitamin D and TBS improvement in rheumatoid arthritis patients after 12 months in both the bDMARDs-treated group (pre and postmenopausal females) and csDMARDS-treated group (only in premenopausal females). Kostoglou-Athanassiou et al. demonstrated that low 25-OH vitamin D serum levels might be associated with the degree of disease activity. 25-OH vitamin D insufficiency leads to high disease activity and generalized musculoskeletal pain [34]. Kwon et al. showed that BMD values significantly increased in RA patients receiving one year of 25-OH vitamin D supplementation [35]. 25-OH vitamin D deficiency directly affects bone microarchitecture, influencing the TBS [8].

RA is an independent risk factor for osteoporosis. Therefore, early, and aggressive treatment with bDMARDs or csDMARDs is mandatory, particularly with anti-TNF α agents, as we have demonstrated in our study by the beneficial effect of these agents on bone metabolism and inflammation [36,37]. It seems that RA patients are highly likely to have 25-OH vitamin D deficiency, and there might be a connection between low 25-OH vitamin D serum levels, disease activity, and bone loss, and that 25-OH vitamin D supplementation is necessary to prevent osteoporosis and the risk of fracture. In addition to 25-OH vitamin D, hyperhomocysteinemia has shown a potential role in bone resorption, as demonstrated by low TBS and BMD values after one year. In determining the risk of fracture in patients with RA, TBS, a novel method for assessing bone microarchitecture, has proven to be more effective than BMD. This study’s major strength is that it paves the road for future research, as data in the literature are scarce. The study limitations are the small sample size and the retrospective study design. Therefore, we believe that prospective studies with larger sample sizes, that analyze the role of anti-TNF inhibitors, homocysteine and 25-OH-vitamin D levels in the trabecular bone score, are necessary for better management of rheumatoid arthritis patients and the complications of this disease.

## 5. Conclusions

In our study, we tried to emphasize the potentially beneficial role of anti-TNF α agents on TBS. This effect seems to be highly correlated with serum 25-OH vitamin D and homocysteine levels. Anti-TNF inhibitors may improve bone mineral density and microarchitecture. Therefore, they can reduce the risk of fractures in patients with RA. Further research is necessary in order to establish the role of these findings in medical practices.

## Figures and Tables

**Table 1:** Demographic characteristics of the study groups, comorbidities, and risk factors of osteoporosis.

	bDMARD Group	csDMARDs Group
Age:		
20–50.1 years	13 (38.23%)	18 (60%)
>50 years	21 (61.76%)	12 (40%)
Menopausal status:		
Premenopausal females	15 (44.12%)	18 (60%)
Postmenopausal females	19 (55.88%)	12 (40%)
RA disease duration (years)	7.61 ± 6.18	5.5 ± 3.36
Alcohol intake	4 (13.6%)	2 (6%)
Smoking status	7 (23.8%)	2 (6%)
Fracture history	0 (0.0%)	0 (0.0%)
Current use of glucocorticoids	0 (0.0%)	0 (0.0%)
Current use of antiosteoporotic		
treatment	0 (0.0%)	0 (0.0%)
Hypertension	15 (5.1%)	5 (15%)
Diabetes mellitus	0 (0.0%)	0 (0.0%)
Cancer	0 (0.0%)	0 (0.0%)
Treatment:		
Infliximab	9 (26.4%)	
Adalimumab	13 (38.23%)	
Etanercept	12 (26.4%)	
Methotrexate		16 (53.3%)
Leflunomide		14 (46.7%)

RA, rheumatoid arthritis; For RA disease duration it was used Mean ± SD (Standard deviation); For the other values it was used the number of cases n (%).

**Table 2:** Characteristics of the first group (34 patients on anti-TNF alpha drugs).

Characteristic	Normal	Abnormal	
**Serum level of homocysteine**	16 (47.1%)	18 (52.9%)-Hyperhomocysteinemia	
T score values at baseline (T1 score)	6 (17.6%)	28 (82.35%)	
		Osteopenia	Osteoporosis
		14 (41.2%)	14 (41.2%)
Lumbar spine TBS at baseline (TBS 1)	2 (5.9%)	32 (94.1%)	
		Slight bone architectural change	Significantly low TBS1 value
		19 (55.8%)	13 (38.2%)
Serum 25-OH vitamin D level	14 (41.2%)	20 (58.8%)-Insufficiency	

**Table 3:** Characteristics of the first group according to menopausal status.

Characteristic	Postmenopausal Females			Premenopausal Females		
**Post/Premenopausal**	19 (55.88%)			15 (44.12%)		
**High level of homocysteine**	11 (73.3%)			7 (57.8%)		
T1 score	Normal	Osteopenia	Osteoporosis	Normal	Osteopenia	Osteoporosis
		6 (31.6%)	13 (68.4%)	6 (40%)	8 (53%)	1 (6.66%)
TBS1	Significantly modified	Slightly modified		Significantly reduced	Slightly modified	Normal
	11 (55.9%)	8 (42.1%)		2 (13.3%)	11 (73.3%)	2 (13.3%)
25-OH vitamin D level	12 (63.1%)–insufficiency/deficiency			8 (53.3%)–insufficiency/deficiency		
T2 score	Normal	Osteopenia	Osteoporosis	Normal	Osteopenia	Osteoporosis
	8 (42.1%)	7 (36.8%)	4 (21.05%)	10 (66.6%)	5 (33.3%)	0 (0%)
TBS2	Normal	Slight degradation	Severe degradation	Normal	Slight degradation	Significant degradation
	2 (13.3%)	10 (52.6%)	7 (36.8%)	5 (33.33%)	10 (66.66%)	0 (0%)

T2 score = T score after 12 months of treatment with anti-TNFα inhibitors.

**Table 4:** Improvements after 12 months according to treatment (bDMARD).

	Treatment		
Characteristics	Etanercept	Infliximab	Adalimumab
Number of pacients	12 (35.3%)	9 (26.5%)	13 (38.2%)
T score	significantly improved	88.8% significant improvement 11.1% no improvement 0% decrease	69.2% improved 30.77% no change
TBS score	50% improved 50% no change	33.3% improved 66.7% no change	61.53% improved 38.46% no change

**Table 5:** Characteristics of the second group (30 patients on csDMARDs drugs).

Characteristics			
**Treatment**	**Methotrexate**	Leflunomide	
	16 (53.33%)	14 (46.67%)	
Menopausal status	Premenopausal	Postmenopausal	
	12 (40%)	18 (60%)	
Age	20–50 years	aged >50 years	
	12 (40%)	18 (60%)	
Clinical characteristic	Normal	Abnormal	
ACPA and RF	6 (20%)	24 (80%)	
Serum level of homocysteine	11 (36.7%)	19 (63.3%)-increased	
T score values at baseline (T1 score)	7 (23.3%)	23 (76.67%)	
		Osteopenia	Osteoporosis
		11 (36.6%)	12 (40%)
Lumbar spine TBS at baseline (TBS 1)	6 (20%)	24 (80%)–low value	
		Slight bone architectural change	Significantly low TBS1 value
		14 (46.6%)	10 (43.7%)
Serum 25-OH vitamin D level	8 (26.7%)	22 (73.3%)-insufficiency (<20 ng/mL)	
T score after 12 months of treatment	favorable evolution	no change	worsening status
	5 (16.66%)	11 (36.66%)	14 (46.66%)

**Table 6:** Characteristics of the second group according to menopausal status.

Characteristic	Premenopausal Women			Postmenopausal Women		
**Post/Pre menopausal**	40%			60%		
**T2 score (%)**	Normal	Osteopenia	Osteoporosis	Normal	Osteopenia	Osteoporosis
	16.67%	58.33%	25%	5.55%	55.55%	38.88%
TBS2	Normal	Slight degradation	Severe degradation	Normal	Slight degradation	Significant degradation
	25%	50%	25%	5.5%	44.4%	50%

T2 score = T score after 12 months of treatment with anti-TNFα inhibitors.

**Table 7:** Improvements after 12 months according to treatment (csDMARDs).

	Treatment					
Characteristic	Methotrexate			Leflunomide		
**No of pacients**	**53.33%**			46.67%		
Evolution after 12 months	Improvement	No change	Decline	Improvement	No change	Decline
T score	12.5%	43.75%	43.75%	21.42%	28.57%	50%
TBS score	70%	30%	0%	0%	57.14%	42.85%

## Data Availability

Datasets generated and/or analyzed during the current study are available from the corresponding authors upon reasonable request.
